# Recent Advances in Understanding Werner Syndrome

**DOI:** 10.12688/f1000research.12110.1

**Published:** 2017-09-28

**Authors:** Raghavendra A. Shamanna, Deborah L. Croteau, Jong-Hyuk Lee, Vilhelm A. Bohr

**Affiliations:** 1 Laboratory of Molecular Gerontology, National Institute on Aging, National Institutes of Health, Baltimore, Maryland, USA

**Keywords:** Werner Syndrome, WRN, DSB repair, Aging, Senescence, Telomere maintenance

## Abstract

Aging, the universal phenomenon, affects human health and is the primary risk factor for major disease pathologies. Progeroid diseases, which mimic aging at an accelerated rate, have provided cues in understanding the hallmarks of aging. Mutations in DNA repair genes as well as in telomerase subunits are known to cause progeroid syndromes. Werner syndrome (WS), which is characterized by accelerated aging, is an autosomal-recessive genetic disorder. Hallmarks that define the aging process include genomic instability, telomere attrition, epigenetic alterations, loss of proteostasis, deregulation of nutrient sensing, mitochondrial dysfunction, cellular senescence, stem cell exhaustion, and altered intercellular communication. WS recapitulates these hallmarks of aging and shows increased incidence and early onset of specific cancers. Genome integrity and stability ensure the normal functioning of the cell and are mainly guarded by the DNA repair machinery and telomeres. WRN, being a RecQ helicase, protects genome stability by regulating DNA repair pathways and telomeres. Recent advances in WS research have elucidated WRN’s role in DNA repair pathway choice regulation, telomere maintenance, resolution of complex DNA structures, epigenetic regulation, and stem cell maintenance.

## Introduction

Werner syndrome (WS) is a segmental progeria. It belongs to a small group of disorders characterized by accelerated aging. WS patients in their 20s and 30s display features similar but not identical to those of normal older individuals, including skin atrophy, graying and loss of hair, wrinkles, loss of fat, cataracts, atherosclerosis, and diabetes (reviewed in Yokote *et al.* ^1^). WS is caused by mutations in the *WRN* gene and has an estimated global incidence ranging between 1 in 1,000,000 and 1 in 10,000,000 births; however, the incidence is higher in Japan at 1 in 100,000 births ^2^. WS is inherited in an autosomal-recessive manner. To date, a total of 83 different mutations in *WRN* have been identified and catalogued by the International Registry of WS (Seattle, WA, USA) and the Japanese Werner Consortium (Chiba, Japan) ^2^. Because of its resemblance to normal aging, WS is widely studied in the field of aging, and many consider WS the best example of an accelerated aging syndrome.

Diagnostic criteria for WS were proposed in 1994 ^3^ and recently updated ^4^. Individuals with WS develop normally until their first decade, and the first clinical sign of the syndrome appears as lack of the pubertal growth spurt during their teen years. Affected individuals in their 20s and 30s begin to manifest skin atrophy and loss and graying of hair. Bilateral cataracts, abnormal glucose and lipid metabolism, hypogonadism, skin ulcers, and bone deformity appear by the fourth decade. Fatty liver, osteoporosis, and calcification of the Achilles tendon are also predominantly observed. WS may be a good model to study sarcopenia ^5^. Malignancy and atherosclerotic vascular diseases such as myocardial infarction are the major causes of death among patients with WS.

The *WRN* gene codes for the WRN protein. WRN is a member of the RecQ helicase family of proteins and is unique in that it possesses both helicase and exonuclease domains ^6^. WRN also has strand annealing activity, but its *in vivo* role remains unclear. Recently, López-Otín *et al.* created a list of pathways that are changed during aging ^7^. These hallmarks of aging pathways have been widely considered the key processes affected during aging. Since WS clinical features include many aspects of normal aging, it is not surprising that WRN functions in, or its loss impacts, many of these pathways. In this review, we survey the literature and compare each aging hallmark against patients with WS ( Table 1). We go on to describe a few key areas of recent WRN-related advances and then point out areas for future research.

**Table 1.** Hallmarks of aging in comparison with Werner syndrome.

Aging hallmarks	Brief description	Werner syndrome (WS)	Reference for WS
**Genome** **instability**	Alteration to the genetic information over time due to DNA damage and defective DNA repair mechanisms. Genomic instability affects overall functions of the cell.	Patient cells show gross genomic instability. WRN-deficient cells display large deletions.	Salk *et al.* ^8^ Chen *et al.* ^9^
**Telomere** **attrition**	Progressive decrease in telomere length over multiple cell divisions. Telomere attrition mainly occurs owing to the end-replication problem and the lack of telomerase enzyme.	WRN interacts with Pot1 and TRF2 components of the shelterin complex to promote telomere maintenance. Telomere length in older patients with WS (40–60 years) is markedly shorter than in younger patients with WS (~30 years) and age-matched non-WS individuals.	Opresko *et al.* ^11^ Ishikawa *et al.* ^10^ Tokita *et al.* ^72^
**Epigenetic** **alterations**	Involves alterations in the DNA methylation patterns, post-translational modification of histones, and chromatin remodeling	Patients with WS show an increased DNA methylation age with an average of 6.4 years. WRN interacts with methylation complex consisting of SUV39H1, HP1α, and LAP2β, which is responsible for the epigenetic histone mark H3K9 trimethylation (H3K9me3). In response to DNA damage, WRN recruits chromatin assembly factor 1 (CAF-1) to alter chromatin structure.	Maierhofer *et al.* ^12^ Jiao *et al.* ^78^ Zhang *et al.* ^14^
**Loss of** **proteostasis**	Impairment of protein homeostasis due to accumulation of misfolded proteins and deregulation of proteolytic system. Chronic expression of misfolded, unfolded, or aggregation of proteins contributes to the development of age- related pathologies such as Alzheimer’s disease and cataracts.	Cataracts are one of the most common features observed in patients with WS. WRN expression is severely affected by promoter hypermethylation in age- related cataract lens cells.	Zhu *et al.* ^15^
**Mitochondrial** **dysfunction**	Reduction in the biogenesis of mitochondria and mitophagy. Reduced ATP production coupled with increased electron leakage. Oxidation of mitochondrial proteins.	WS cells show increased reactive oxygen species (ROS) production. Hepatocytes of Wrn (Δhel/Δhel) mice have decreased mitochondria and show altered mitochondrial functions.	Cogger *et al.* ^16^
**Cellular** **senescence**	Stable arrest of the cell cycle coupled with stereotyped phenotypic changes such as the accumulation of persistent DNA damage, senescence-associated β-galactosidase, p16 ^INK4A^, and/or telomere shortening	Cellular senescence is a striking feature of WS patient cells. WRN deficiency increased the accumulation of persistent DNA damage, p16, and senescence- associated β-galactosidase.	Norwood *et al.* ^73^ Lu *et al.* ^13^
**Deregulated** **nutrient sensing**	Somatotropic axis essentially consisting of growth hormone, insulin-like growth factors (IGF-1 and II), and their carrier proteins and receptors regulates metabolism in mammals. In addition to insulin–IGF-1 (IIS) signaling pathway, which senses glucose, three interconnected nutrient sensing systems are associated with aging. The mechanistic target of rapamycin (mTOR) senses high amino acid concentrations, AMPK (5′- adenosine monophosphate [AMP]-activated protein kinase) senses low-energy states by detecting high AMP levels, and sirtuins sense low-energy states by detecting high NAD ^+^ levels. With aging, IIS pathway decreases, mTOR activity increases, AMPK upregulates in skeletal muscles, and sirtuins are downregulated.	WRN protects against starvation-induced autophagy. Further research is required to elaborate the role of WRN in regulating nutrient-sensing mechanisms.	Maity *et al.* ^17^
**Stem cell** **exhaustion**	A decline in the proliferation of stem and progenitor cells, which are required for tissue regeneration	WRN-deficient mesenchymal stem cells showed progressive disorganization of heterochromatin and premature senescence.	Zhang *et al.* ^14^
**Altered** **intercellular** **communication**	Enhanced activation of nuclear factor kappa B (NF-κB) and increased production of tumor necrosis factor (TNF), interleukin-1 beta (IL-1β), and cytokines resulting in age-associated alteration in intercellular communication. Accumulation of pro-inflammatory tissue damage, failure of immune system to clear pathogens and dysfunctional host cells, and occurrence of defective autophagy response. Bystander effect in which senescent cells induce senescence in neighboring cells via gap junction–mediated cell-cell contacts and ROS.	Patients with WS have elevated levels of inflammation-driven aging- associated cytokines (IL-4, IL-6, IL-10, granulocyte macrophage colony- stimulating factor [GM-CSF], IL-2, TNF- α, interferon gamma [IFNγ], monocyte chemoattractant protein-1 [MCP-1], and granulocyte colony-stimulating factor [G- CSF]) compared with normal individuals.	Goto *et al.* ^18^

Aging research has enumerated nine hallmarks of aging: genomic instability, telomere attrition, epigenetic alterations, loss of proteostasis, deregulated nutrient sensing, mitochondrial dysfunction, cellular senescence, stem cell exhaustion, and altered intercellular communication ( Table 1) ^7^. Patients with WS have defects in DNA repair machinery and show genomic instability ^8, 9^. WRN, in association with the telomere-protecting shelterin complex, promotes telomere maintenance, and loss of WRN, as seen *in vitro* and in patients with WS, results in the rapid decline of telomere length ^10, 11^. A progressive increase in DNA methylation is considered an aging biomarker, and, consistent with this, patients with WS display increased epigenetic age ^12^. Increased DNA damage accumulation, genomic instability, telomere attrition, and histone methylation are contributing factors for cellular senescence and stem cell exhaustion in WS ^13, 14^. Although extensive research is required to sort out the molecular functions of WRN in regulating proteostasis, nutrient sensing, and mitochondria, WS is phenotypically associated with a loss in proteostasis and mitochondrial dysfunction ^15, 16^. WRN protects cells from starvation-induced autophagy, which is deregulated by an imbalance in nutrient-sensing mechanisms ^17^. Inflammation alters intercellular communication owing to the accumulation of cytokines increasing with aging. Patients with WS have elevated cytokine levels of interleukin-2 (IL-2), IL-4, IL-6, tumor necrosis factor alpha (TNF-α), interferon gamma (IFNγ), and monocyte chemoattractant protein-1 (MCP-1) ^18^.

In the past five years, there have been a large number of studies on WS, covering areas including those discussed in Table 1. Here, we discuss some areas of particular relevance where significant insight has been gathered in recent years. These include the role of WRN in DNA double-strand break (DSB) repair, telomere maintenance, senescence and heterochromatin stabilization, and cancer. We will discuss these selected areas in depth below.

## WRN regulates double-strand break repair pathway choice

The human genome is under constant exposure to exogenous and endogenous agents. DSBs are among the most potent and deleterious forms of cellular DNA damage, causing mutagenic changes, developmental defects, gross chromosomal rearrangements, cell death, and malignancy ^19^. Approximately 10 to 50 DSBs are being formed per cell per day ^20^. DSBs are mainly detected, processed, and repaired by two pathways: homologous recombination (HR) and non-homologous end joining (NHEJ). The choice of DNA repair pathway is tightly regulated and associated with the cell cycle. While NHEJ is active throughout the cell cycle, DSBs in S and G _2_ phases are preferably repaired by HR using the intact sister chromatid. WRN recruits to DSB sites in G _1_ as well as in S and G _2_ phases ^21^. The DSB sensor protein complexes Ku70/80 and MRN (MRE11, RAD50, NBS1) initiate NHEJ and HR pathways, respectively. The HR pathway is a high-fidelity DNA repair mechanism. In contrast, the NHEJ pathway is an error-prone mechanism where the DSBs are processed and ligated without relying on sequence homology ^22^. Despite its error-prone nature, NHEJ is the predominant form of DSB repair in human somatic cells. In addition to these DSB repair pathways, error-prone alternative (alt)-NHEJ and single-strand annealing also operate under various conditions ^22^.

WRN mainly localizes to the nucleolus, and then translocates to DSBs, when introduced. The acetylation of WRN by CBP/p300 affects its subcellular distribution, and deacetylation mediated by Sirt1 affects its translocation to the nucleolus ^23, 24^. In response to DNA damage, WRN interacts with several proteins that participate in HR, NHEJ, and single-strand annealing ^6^. Recent advances in DSB repair suggest the existence of two distinct mechanisms of NHEJ: classical/canonical (c)-NHEJ and alt-NHEJ ^25^. Alt-NHEJ is distinguished from c-NHEJ by the participating proteins, DSB resection, and the use of microhomology during end joining. The essential factors involved in c-NHEJ include Ku70/80 heterodimer, DNA-dependent protein kinase catalytic subunit (DNA-PKcs), and XRCC4/ligase IV (X4L4) complex. Alt-NHEJ mainly acts as a backup pathway to c-NHEJ and operates as a major pathway of DSB repair in Ku70/80-deficient cells and ligase IV-deficient cells ^26, 27^. Alt-NHEJ depends on proteins that participate in HR; however, the pathway does not depend on a homologous sister chromatid. MRE11, PARP1, CtIP, DNA ligase I, and DNA ligase III all promote alt-NHEJ ^28– 30^. Research from our lab and others identified physical interactions of WRN with Ku70/80, DNA-PKcs, X4L4, PARP1, DNA ligase I, DNA ligase III, and MRN ^31– 37^.

The Ku70/80 heterodimer in association with DNA-PKcs initiates a cascade of events that constitutes the c-NHEJ pathway ^38^. The Ku70/80 complex interacts directly with WRN and stimulates its exonuclease activity ^31, 39^. WRN has two putative Ku-binding motifs, one in the N-terminus and another in the C-terminus, which accelerate DSB repair. The N-terminal Ku-binding motif mediates Ku-dependent stimulation of WRN exonuclease activity ^40^. DNA-PKcs, which gains robust kinase activity by interacting with DSB-bound Ku70/80, phosphorylates WRN at S440 and S467 positions and regulates WRN’s enzymatic activities ^32, 41, 42^. With its nuclease activity, WRN processes DNA ends and generates substrates suitable for ligation mediated by the X4L4 complex ^33^. Ku-mediated c-NHEJ dominates over all other DSB repair pathways, while alt-NHEJ is the default DNA repair pathway in Ku-deficient cells or under conditions that inhibit c-NHEJ ^43^. WRN’s role in DSB repair pathways is complex; however, findings clearly demonstrate that WRN stimulates c-NHEJ with its enzymatic activities and inhibits alt-NHEJ with its non-enzyme functions ^21^.

**Figure 1.**
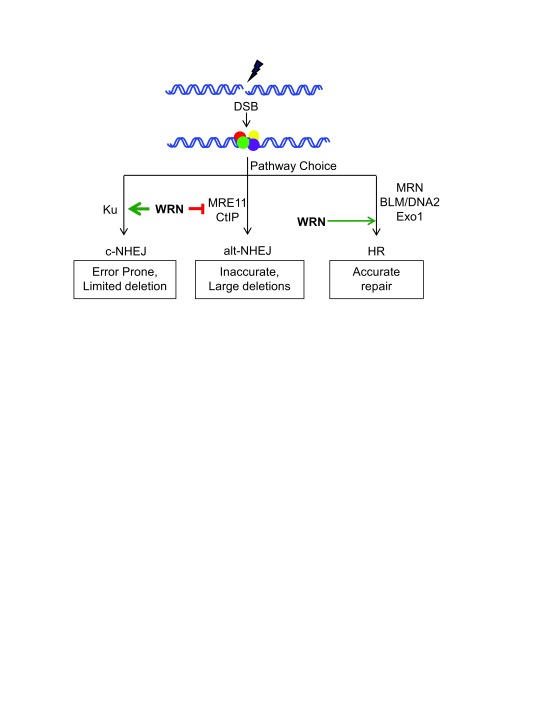
Double-strand break (DSB) repair pathway choice. DSBs generated by extrinsic and intrinsic factors are recognized by the sensor proteins Ku70/Ku80, WRN, MRN, and PARP1 to mediate repair. DSBs are repaired via classical/canonical non-homologous end joining (c-NHEJ), alternative (alt)-NHEJ, and homologous recombination (HR) pathways. WRN promotes Ku-dependent c-NHEJ with its catalytic activities and strongly inhibits alt-NHEJ with its non-catalytic activities. WRN suppresses the recruitment and downstream functions of MRE11 and CtIP to inhibit alt-NHEJ. During S/G _2_ phases of the cell cycle, WRN promotes HR. Accurate repair of DSBs is required for genome stability without loss of genetic information.

The accurate repair of DSBs depends on the regulation of end processing. Resection of DSBs is very limited during c-NHEJ and is extensive during HR and alt-NHEJ. End resection is carried out in a two-step process: initial resection (short-range), which is regulated by MRN complex with CtIP, and extended resection (long-range) performed by DNA2/BLM or EXO1 ^44^. End resection during HR and alt-NHEJ is initiated by MRE11 in association with CtIP. Interestingly, WRN actively restrains 5′-3′ end resection by inhibiting the recruitment of MRE11 and CtIP to DSBs, specifically in G _1_ phase. Consequently, alt-NHEJ is upregulated in WS cells and WRN-deficient cells, resulting in telomere fusions. Consistent with this finding, the inhibition of alt-NHEJ by downregulation of CtIP suppresses telomere fusions in WRN-deficient cells ^21^. WRN has also recently been shown to inhibit MRE11/Exo1-dependent end resection and generation of single-stranded DNA in camptothecin (CPT)-treated cells ^45^. CPT is an anti-cancer agent which blocks replication and induces WRN degradation ^46, 47^. Replication fork progression in CPT-treated (low-dose) WS cells was rescued by expressing wild-type WRN ^42^, and the exonuclease activity of WRN was required to protect DSBs at replication forks from MRE11-dependent processing ^45^. WRN and DNA2 physically interact with each other and coordinate their enzyme activities to promote double-stranded DNA degradation and resection ^48, 49^. Interestingly, the phosphorylation of WRN at S1133 by cyclin-dependent kinase 1 (CDK1), which occurs during late S/G _2_ and M phases, regulates DSB repair pathway choice between HR and NHEJ ^50^. Taken together, WRN plays a major role in DSB repair pathway choice ( Figure 1).

## Telomere maintenance

Chromosome ends, the telomeres, are unique DNA structures that must be replicated and protected. Human telomeres are approximately 11–15 kilobases in length and composed of about 2,500 repeats of TTAGGG sequence followed by a single-stranded 3′ tail region of the same sequence. With age, telomere length is reduced mainly owing to end-replication problems. Telomeres are packed into protein-DNA complexes with the aid of shelterin proteins. We and others have previously shown that WRN interacts with TRF1, TRF2, and POT1 components of the shelterin complex ^11, 51– 53^.

Several lines of evidence suggest that telomere dysfunction contributes to WS pathology. Cells from WS patients and WRN-deficient cells undergo early replicative senescence and display telomere loss and chromosomal rearrangements. Telomere fusions and chromosome translocations are also well documented in WS patient and WRN-deficient mouse cells ^54– 58^. Importantly, re-introduction of telomerase activity into WS cells prevents senescence and telomere loss ^54, 59^. Additionally, although the WRN protein is ubiquitously expressed, WS patient cells preferentially display premature aging of mesenchymal cells ^60^. Reprogramming of induced pluripotent WS stem cells has reinforced the importance of the roles WRN plays in telomere maintenance because differentiation into any cell which naturally expresses telomerase extends the proliferative capacity of WS cells ^61^. A Wrn-null mouse model further substantiates the importance of WRN in telomere maintenance. These mice failed to show significant pathology until bred with late-generation telomerase-deficient mice, demonstrating that short telomeres were critical to revealing WS-like premature aging features ^62, 63^.

WRN acts at telomeres to promote replication and suppress recombination. Cells use telomerase, a reverse transcriptase enzyme with an RNA component, Terc, to replicate and lengthen telomeres. It is thought that WRN’s helicase activity contributes to telomere replication through the resolution or dissolution of complex DNA structures found at telomeres such as T-loops, D-loops, and G-quartets (G4s) ^11, 64– 66^. G4s are formed by four guanines associated through Hoogsteen base pairing. They are thought to arise in areas of single-stranded DNA, in regions undergoing replication and transcription, and preferentially in the telomeric G-rich strand. G4s may promote genomic instability; therefore, enzymes, like WRN, exist to unwind them, thereby suppressing recombination ^66– 69^.

In the absence of telomerase, cells maintain their telomeres via recombination mechanisms termed alternative lengthening of telomeres (ALT). ALT cells and cells without WRN protein show increases in telomere-sister chromatid exchanges ^21, 56, 70^. This increase has been, in part, attributed to a rise in alt-NHEJ in WRN-deficient cells ^21^. Interestingly, knockdown of WRN in three different ALT cell lines demonstrated variable dependence on WRN to prevent telomere loss ^71^. This suggests that there are multiple telomerase-independent mechanisms that contribute to telomere maintenance.

Although it is routinely reported that skin cells from patients with WS have shorter telomere length ^10^, it is still debated whether this is true in all organs and how it contributes to the pathology found in patients with WS. In one study of two patients with WS at autopsy, the authors did not find substantially shorter telomeres from the liver relative to controls ^72^. However, the liver is considered a regenerative organ and therefore may have the capacity to re-activate telomerase in this tissue, negating the impact of WRN loss. In another recent study, younger WS patients with intractable ulcers had normal terminal restriction fragment lengths, suggesting that telomere length was not likely driving this phenotype ^10^. While the prevailing theory is that telomere-driven replicative senescence promotes pathology in WS, this may need to be revised as we learn more about telomeres in different organs from patients with WS.

## Epigenetic modification and senescence

*In vitro*, premature cellular senescence is a striking feature of WRN-deficient cells ^73^. Senescent cells are defined as viable growth-arrested cells. Senescence and exhaustion of stem cells are thought to contribute to tissue degeneration and aging. There are many actions which induce cellular senescence: extended cellular division, oncogene activation, telomere attrition, and exposure to DNA-damaging agents ^74^. Senescent cells typically have an altered appearance and elevated secretion of pro-inflammatory cytokines which promote inflammation ^75^. Until recently, cellular senescence in WRN-deficient cells was believed to be due to telomere issues and the accumulation of replication-associated endogenous DNA damage.

The genome-wide distribution of histone methylation marks changes during aging ^76^. Consistent with WS as an aging model, patients with WS display increased epigenetic age as measured by DNA methylation of known aging biomarkers ^12^. In humans, H3K9 trimethylation (H3K9me3) denotes constitutive heterochromatin and is mainly methylated by SUV39H1/2 histone methyltransferase (HMTase) ^77^. Though not fully characterized, loss of heterochromatin is considered to increase the susceptibility of genomic DNA to mutations and reduce transcriptional precision, both of which promote genomic instability during aging ^76^. Interestingly, Zhang *et al.* ^14^ reported that WRN exists in complex with SUV39H1, HP1α, and LAP2β, which together are responsible for the epigenetic histone mark H3K9me3. WRN also interacts with the chromatin remodeling chaperone chromatin assembly factor 1 (CAF-1) ^78^, which deposits histones H3 and H4 onto newly replicated DNA ^79^. In response to DNA damage, WRN recruits CAF-1 and participates in chromatin structure restoration ^78^.

In stem cells, the histone methylation pattern is preserved over generations and is associated with the maintenance of stem cell potential. Mesenchymal stem cells differentiated from WRN-null embryonic stem cells display genome-wide reductions in H3K9me3 levels contributing to heterochromatin disorganization and stem cell exhaustion ^14^. Specifically, the loss of WRN resulted in loss of heterochromatin in subtelomeric and subcentromeric regions and altered the transcription of repetitive satellite DNA. Since epigenetic changes are a known hallmark of aging, Zhang *et al.* tested whether disorganized heterochromatin could induce cellular senescence ^14^. Disorganized heterochromatin, without concomitant DNA damage, does in fact induce cellular senescence, and restoration of heterochromatin suppresses the senescence phenotype. Thus, the authors suggested that WRN participates in heterochromatin stability and demonstrated that heterochromatin disorganization is yet another mechanism whereby WRN deficiency can promote cellular senescence and aging.

Attempts to safely destroy senescent cells is a burgeoning field and one in which patients with WS may derive benefits. Since replicative senescence is a classic feature of WS cells, several groups are using WS cells to identify and characterize anti-senescent agents. An inhibitor of p38, a mitogen-activated protein kinase, improved replication capacity in WS fibroblasts ^80, 81^. Rapamycin, a mechanistic target of rapamycin (mTOR) kinase inhibitor, decreased 53BP1 foci, a marker for DNA damage, and increased proliferation in WRN-knockdown cells after long-term treatment ^82^. Rapamycin and hydrogen sulfide both have been shown to decrease senescence in WS patient cells, perhaps through Sirt1, although this needs further investigation ^83^. Translational applications developed from WRN-deficient systems may not only benefit patients with WS but also provide insight into basic mechanisms of aging.

## WRN in cancer research

WRN promotes DNA repair and genome stability, and consequently patients with WS are predisposed to various cancers. The most common neoplasia in patients with WS are thyroid cancer, malignant melanoma, meningioma, soft tissue sarcoma, osteosarcoma, breast cancer, and leukemias ^84, 85^. Additionally, there is a higher prevalence of mesenchymal or non-epithelial malignancies (that is, so-called sarcomas) in patients with WS in contrast to normal older individuals who usually develop malignancies of epithelial origin. It is widely believed that *WRN* functions as a tumor suppressor gene. Although patients with WS develop various cancers, limited studies have been conducted to correlate WRN mutations in cancers in non-WS individuals. Interestingly, race-specific mutations in *WRN* were found to be associated with increased breast cancer risk. Breast cancer risk was increased by the Cys1367Arg mutation in German and Austrian populations and by the Phe1074Leu mutation in Taiwanese and Chinese populations ^86– 89^.

Primary tumors of colorectal cancer patients and cell lines display decreased WRN mRNA and protein expression ^90, 91^; however, no WRN mutations have been associated with colorectal cancer risk ^92, 93^. Irinotecan (CPT-11), a semi-synthetic derivative of CPT, which is routinely used in colorectal cancer therapy, enhances the survival of colorectal cancer patients with hypermethylated WRN promoter ^90^. In contrast, Bosch *et al.* reported that hypermethylation of the WRN promoter does not have predictive value for personalized irinotecan-based therapy ^91^. The observed differences could be due to the complex nature of promoter hypermethylation and varied expression of WRN. CPT and its derivatives inhibit DNA topoisomerase I (Top1) and generate DSBs during replication. WRN physically and functionally interacts with Top1, and WRN helps in resolving CPT-induced DNA lesions. Furthermore, the ectopic expression of WRN inhibits CPT-induced cellular senescence, cell death, and enhanced replication fork recovery and DSB repair ^45, 46, 48^. Interestingly, recent studies suggest that CPT induces WRN and Top1 degradation via the ubiquitin proteasome pathway ^46, 47^. CPT-induced WRN degradation, but not Top1 degradation, was found specifically in CPT-sensitive cells ^30^. Thus, it is possible that WRN expression or degradation (or both) could be used as a biomarker for personalized chemotherapy, and further research should explore this potential.

## Conclusions and future perspectives

**Figure 2.**
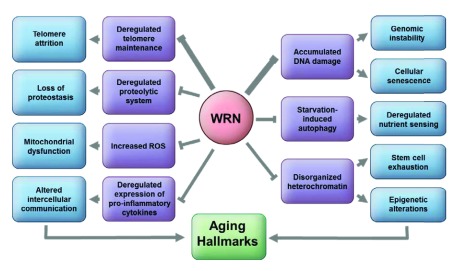
The role of WRN in the contexts of the known hallmarks of aging ^7^. The widths of the lines showing inhibition indicate the estimated relative involvement of WRN in the processes. ROS, reactive oxygen species.

As shown in Table 1, many of the hallmarks of aging are found in patients with WS and altered as a direct consequence of WRN loss. Although there is strong evidence for a role for WRN in several of the pathways ( Figure 2), others show a weak association and need further investigation. Patients with WS display many aging features, but the initiating pathology for most is still not known. For example, patients with WS suffer from severe intractable foot ulcers; however, the underlying pathology has yet to be understood. Cataracts are another cardinal feature found in patients with WS, but the mechanism or mechanisms instigating cataracts in patients with WS have to be identified. Age-associated cataracts occur because of an imbalance in the proteostasis in the lens cells ^94^; perhaps a similar mechanism is at play in WS. Recent studies suggest that WRN regulates cellular functions, like DSB repair, via catalytic and non-catalytic functions. Further investigations into its catalytic and non-catalytic functions may help elucidate disease pathology. Additional research is also needed to further define the specific functions for the exonuclease and helicase domains and to understand why these two activities are present in the same protein. They may cooperate in some pathways such as base excision repair ^95^ or telomere maintenance ^11^ or they may not. Model organisms may be of benefit in this research because not all species express both the helicase and the exonuclease from one gene. In *Caenorhabditis elegans* and *Drosophila*, the two domains are expressed from separate genes. Additionally, the analysis of WRN functions in model organisms will help identify conserved and divergent WRN roles over an organism’s life span.

Mammals have five RecQ helicases; it is important to dissect out why and how they cooperate in genome maintenance. Mutations in three of the five human RecQ helicases cause unique syndromes, indicating that they have non-overlapping functions. Patients with WS are affected by certain types of cancers compared with patients with Bloom and Rothmund-Thomson syndrome. Studies identifying the mechanisms behind the susceptibility of these patients to certain type of cancers are still needed. Furthermore, the reason why patients with WS are prone to non-epithelial malignant tumors remains to be determined.
